# Correlation between neural responses and human perception in figure-ground segregation

**DOI:** 10.3389/fnsys.2022.999575

**Published:** 2023-01-12

**Authors:** Motofumi Shishikura, Hiroshi Tamura, Ko Sakai

**Affiliations:** ^1^Department of Computer Science, University of Tsukuba, Tsukuba, Japan; ^2^Graduate School of Frontier Biosciences, Osaka University, Suita, Japan; ^3^Center for Information and Neural Networks, Suita, Osaka, Japan

**Keywords:** figure-ground segregation, V4, population coding, physiological experiment, psychophysical experiment

## Abstract

Segmentation of a natural scene into objects (figures) and background (ground) is one of crucial functions for object recognition and scene understanding. Recent studies have investigated neural mechanisms underlying figure-ground (FG) segregation and reported neural modulation to FG in the intermediate-level visual area, V4, of macaque monkeys (FG neurons). However, whether FG neurons contribute to the perception of FG segregation has not been clarified. To examine the contribution of FG neurons, we examined the correlations between perceptual consistency (PC), which quantified perceptual ambiguity in FG determination, and the reliability of neural signals in response to FG. First, we evaluated PCs for the images that were used in the previous neural recording in V4; specifically, we measured how consistently FG can be determined across trials and participants for each stimulus. The PCs were widely distributed, so that we identified the ambiguity in FG segregation for each stimulus. Next, we analyzed the correlation between the PCs and the reliability of neural modulation to FG. We found that the stimuli with higher PCs evoked more consistent and greater modulation in the responses of single neurons than those with lower PCs. Since perception is expected to show a greater correlation with responses of neural population compared to those of single neurons, we examined the correlation between the PCs and the consistency of the population responses in FG determination. Stimuli with higher PCs evoked higher population consistency than those with lower PCs. Finally, we analyzed the correlation between the PCs and neural latencies in FG modulation. We found that the stimuli with higher PCs showed shorter reaction times in FG perception and evoked shorter modulation latencies in FG neurons. These results indicate that the responses of FG neurons recorded from macaque monkeys show significant correlations with human FG perception, suggesting that V4 neurons with FG-dependent responses contribute to the perception of FG segregation.

## 1. Introduction

The ability to segregate an object (figure) from the background (ground) is crucial in the perception of the position and shape of objects in visual scenes, which is a fundamental function leading to object recognition and scene understanding. Neural responses associated with visual scene segmentation have long been investigated in the ventral visual pathway. Figure-ground (FG) modulation has been reported in V1 and V4 wherein the responses of neurons differed depending on whether a figure or ground was projected onto the classical receptive fields (CRF; e.g., Lamme, 1995; Poort et al., 2016). Specifically, FG modulation was observed depending on whether a texture element was located in a square-shaped figure or outside (ground). Border ownership-selectivity (BOS) has also been reported in V2 and V4 wherein the responses of neurons differed depending on the direction of figure with respect to the border that passed through the center of the CRF (e.g., Zhou et al., 2000; Franken and Reynolds, 2021). Neurons in V4, an intermediate-level visual area, have been reported to exhibit neural modulation in response to stimulus properties such as curvature, closure, and textures, in addition to FG and BOS, and considered to establish the extent of a figural region, which is often called surface construction or a proto-object (Rensink and Enns, 1995; Sajda and Finkel, 1995; Mihalas et al., 2011; Roe et al., 2012; Von der Heydt, 2015; Oleskiw et al., 2018).

Although global configuration and knowledge are generally necessary for FG segregation in natural scenes, a variety of local clues, such as convexity, closedness, and symmetry, also play crucial roles in the segregation (Harrower, 1936). It is not fully understood whether the neurons with FG-dependent modulations are, in fact, the neural substrate underlying the perception of FG. Yamane et al. (2020b) examined the responses of V4 neurons of monkeys to local natural images and reported that approximately one-quarter of the neurons showed significant modulation in firing rates depending on whether a figure or ground region was projected onto the CRF (FG neurons). The responses to hundreds of local natural images were pooled to cancel out the properties of regions including shapes and textures (refer to section “2.3.1. Neurons with FG-dependent responses” for details). Their results also showed that individual neurons exhibited a low consistency in FG discrimination across a wide variety of contours and textures, i.e., figure-preferring neurons sometimes exhibit strong responses to ground depending on the stimuli. This low consistency led us to expect that particular stimuli evoke correct responses of neurons, but others do not, and if so, this property would account for different degrees of perceptual ambiguity in FG segregation depending on stimuli.

**FIGURE 1:**
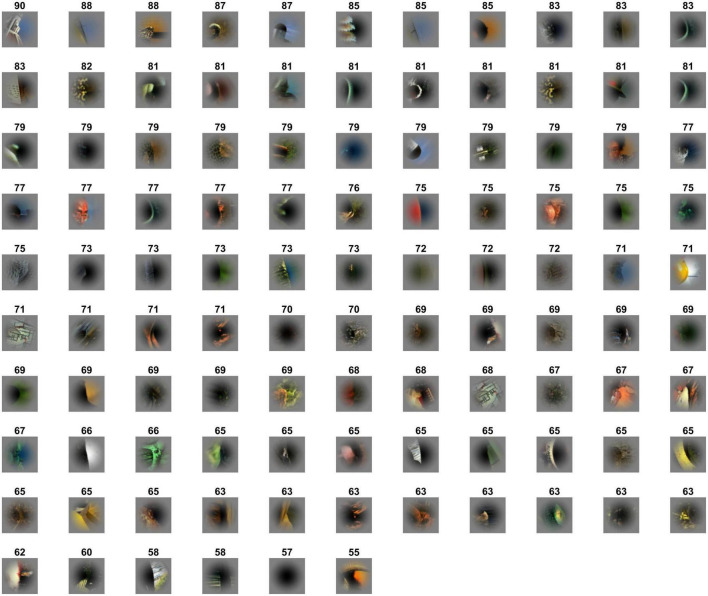
Local natural scene stimuli cropped from the Berkeley Segmentation Dataset. Stimulus patches were cropped from the Berkeley Segmentation Dataset (BSD) such that the contour of an object passed through the center of the patch. The patches are placed in the rank order of perceptual consistency (the numbers indicate the PC values of the patches); the top-left and bottom-middle with the highest and lowest consistencies, respectively. The patches are rotated so that the figure region is located on the left.

Psychophysical studies have reported that the perceptual ambiguity varies across patches. Recent psychophysical studies have reported that image properties such as local contours and textures provide clues for FG perception (Ramenahalli et al., 2014; Sakai et al., 2015), and that the ambiguity in FG perception varies across patches depending on the image properties in local patches (Fowlkes et al., 2007). Humans are often able to perceive figure and ground in local natural images without global information, while some local images appear ambiguous and difficult to judge. For instance, in Figure 1, the figure regions appear easy to find in the upper-left patches compared with those in the lower-right patches. The highly ambiguous image patches show low consistency in FG judgement across trials. This perceptual consistency (PC) in FG determination is expected to originate from the reliability of neural signals underlying FG segregation. We expected that stimuli that allow humans to consistently perceive the figure tend to evoke substantial FG modulation in V4 neurons; the PC in FG judgement is expected to correlate with FG modulation in neural responses.

To better understand the neural correlates underlying FG perception, we investigated the relationship between human FG perception in local natural images and FG modulation in monkey V4 neurons in response to the local images as measured in previous electrophysiological experiments (Yamane et al., 2020b). First, we performed a human psychophysical experiment with the same natural patches as those used in the neural recording to estimate PC for single patches in FG judgments. We compared the estimated PC with the neural consistency that indicates the ratio of correct responses across all trials. Stimuli with higher PC were expected to evoke higher neural consistencies. Second, we investigated the contribution of the population responses of FG neurons to FG perception. Specifically, we compared them with population-based neural consistencies across trials that were estimated from the integration of the responses of a few tens of neurons. A positive correlation between the two would support the link between the population responses and perception. Stimuli with higher PC were expected to evoke higher population-based neural consistencies. Third, we examined correlation between PC and the magnitude of neural FG-modulation. Stimuli with higher PC were expected to show greater neural modulation. Finally, we examined the relation between the reaction time for FG perception and neural latency. Stimuli with higher PCs were expected to exhibit shorter reaction times and evoke shorter neural latencies. The results showed that stimuli with higher PC evoked more consistent responses and greater FG modulation in V4 neurons. The stimuli with higher PC also exhibited shorter reaction times and neural latencies. These results indicated that the responses of V4 neurons with FG-dependence show significant correlations with human FG perception, suggesting that V4 neurons with FG-dependent responses contribute to perceptual FG segregation.

## 2. Materials and methods

### 2.1. Visual stimuli

**FIGURE 2:**
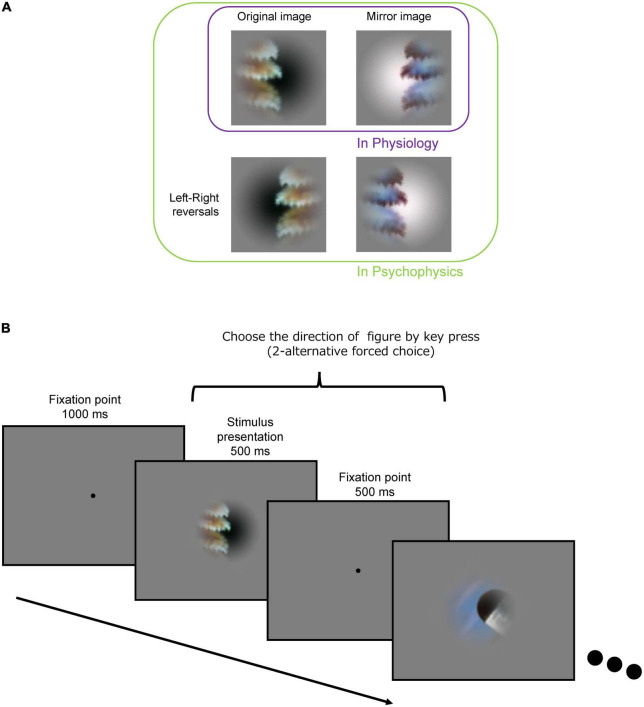
Procedure for the psychophysical experiment. **(A)** Original (top-left), mirror image (top-right), and its left-right reversals (bottom-left and bottom-right) of an example stimulus. **(B)** Following an initial fixation period of 1,000 ms, a stimulus presentation period (500 ms) and a fixation period (500 ms) were repeated. Subjects were asked to indicate the DoF with the left and right arrow keys during the stimulus presentation and fixation periods.

The set of local natural image patches used in the present psychophysical experiments is identical to that used in the previous neural recordings of macaque monkeys (Yamane et al., 2020b). The stimulus set comprised 105 small natural images that were locally cropped from the Berkeley Segmentation Dataset (BSD) (Fowlkes et al., 2007). Example stimuli are shown in Figure 1. Since the contours were cropped so that they passed through the center of the patch, each stimulus patch contained both figure and ground regions. Since the distribution of contour curvature is non-uniform in natural scenes, we controlled the distribution of contour convexity, orientation, closedness, and symmetry (chosen uniformly from each range of these properties) (Sakai et al., 2015). Mirror stimuli were also prepared, which were the mirror images with respect to the tangent of the contour at the center of the original patch; the colors of the mirror images were inverted such that the polarity of the color contrast was constant with respect to the boundary (refer to the top-right image in Figure 2A). Note that in the psychophysical experiments, we also tested the left-right reversals of the original and mirror stimuli (the bottom row in Figure 2A). We have inverted the hue in RGB space (255 – original value for each RGB channel). Thus, the difference in color and luminance between figure and ground was constant but Michelson contrast was not. The mean difference in Michelson contrast between the original and mirror stimuli across the stimuli was 0.026 (standard deviation (SD) = 0.025). It might affect the interpretation of present results but any effect of the difference in contrast appeared to be minimal if existed. A Gaussian function was used to attenuate the contrast toward the periphery to blur the boundary between the patch and the gray background.

### 2.2. Psychophysical experiment

We performed a psychophysical experiment to estimate PC in FG perception. PC represents how consistently humans perceive a figure region in a particular stimulus. With localized or occluded natural images, FG judgments vary across trials and participants.

#### 2.2.1. Procedure

The procedure of the experiment is illustrated in Figure 2B. For an initial fixation, a black dot (fixation point) was presented at the center of display for 1,000 ms. A stimulus patch was presented for 500 ms around the fixation point, followed by presentation of the fixation point for 500 ms. The stimuli (500 ms) and interstimulus fixation (500 ms) were repeatedly presented. The size of the stimulus patch was 6° × 6° in visual angle. The participants indicated the direction of the figure (DoF) with respect to the fixation point at the center of screen by using the left/right arrow keys (two-alternative forced-choice responses) on the keyboard within 1,000 ms after stimulus onset. We instructed the participants to answer in the direction viewed from the fixation point in the foreground and explained foreground is “figure” in the experiment. We have also instructed the participants to respond as fast as possible after stimulus onset. The stimulus patches were rotated and presented so that the participants could answer the direction with left/right keys, i.e., a stimulus was rotated with respect to the center so that the tangent of the contour passing through the center was vertical (0°). We confirmed that this rotation did not affect the present results (refer to Supplementary Figure 1). We also presented the stimuli with the left and right sides reversed (left-right reversal; the bottom images in Figure 2A) to cancel out a possible bias in the preference of the DoF. A total of 105 stimuli were presented in a single session. A total of eight sessions were performed wherein each of eight variant-trials of individual stimuli was presented: the original and mirror stimuli (2), left-right reversals (2), and reversed-order presentations (2) (refer to Figure 2A). Each variant of each stimulus was presented once. The number of participants was ten resulting in a total of 80 trials for each stimulus (8 variants × 10 participants). The order of stimulus presentation was pseudorandomized within the odd-numbered sessions and reversed in the subsequent even-numbered sessions.

#### 2.2.2. Apparatus and participants

The stimuli were displayed on an LCD monitor (PHILIPS 436M6VBPAB; 42.5 inch, 3840 × 2160) with a refresh rate of 59 Hz. The luminance was 0.274 cd/m^2^ for black and 371.0 cd/m^2^ for white. The display was linearized with γ = 2.2. All experiments were conducted in a dark room. The stimulus images were presented on the display using Python and Psychopy (Peirce et al., 2019). Prior to the experiments, 10 individuals with normal or corrected-to-normal visual acuity who were not aware of any color deficiency gave written informed consent to participate in the experiments (age 21–26 years, 3 females and 7 males). All experiments were approved by the Research Ethics Committee of the Faculty of Engineering, Information, and Systems at the University of Tsukuba and were performed in accordance with the approved guidelines.

### 2.3. Electrophysiological experiment

The electrophysiological experiments were previously performed at Osaka University and reported elsewhere (Hasuike et al., 2015; Yamane et al., 2020b). We analyzed the previously recorded neural activities of three hemispheres of two female macaque monkeys (*Macaca fuscata*) provided by the National BioResource Project (MEXT, Japan^1^). Two macaque monkeys were anesthetized and immobilized, and were subjected to the electrophysiological experiments. The stimulus images were presented on a display linearized with γ = 2.2. The recorded data were available in the institutional archive (Yamane et al., 2020a). All animal experiments were performed in accordance with the guidelines of the National Institute of Health (1996) and the Japan Neuroscience Society and were approved by the Osaka University Animal Experiment Committee (certification no: FBS-13-003). The details of the animal welfare and preparation, recording, visual stimuli, and experimental design were described by Yamane et al., 2020b; essential information of the experiments was summarized in this section. The stimuli identical to those used in our psychophysical experiments were shown to the animals, and the spiking activities of V4 neurons were recorded. Single-unit spiking activities were sorted offline for each session. The details of the spike sorting are described elsewhere (Tamura et al., 2014). Note that the stimuli were not rotated as in the psychophysical experiment.

#### 2.3.1. Neurons with FG-dependent responses

FG neurons were defined as neurons that showed FG-dependent responses. Specifically, responses were considered FG-dependent if they occurred at a significantly higher rate when a figure/ground region was projected onto their CRF center compared to when the ground/figure was projected using all the original and mirror stimuli [105 stimuli × 2 (original and mirror)] (F-/G-preferring neurons). The definition of FG modulation is not independent of BOS, and the modulations based on BOS and FG are often indistinguishable (see the detail of the difference between BOS and FG modulation in Supplementary Figure 2). We controlled color contrast by using the mirror stimuli so that the responses to figure and ground with the same color contrast were recorded (e.g., a particular shape was tested with the figures in red and green). We did not control texture since the exchange of textures between figure and ground was not practical with natural images, although some studies using artificial textures (oriented bars inside a square-shaped region surrounded by the orthogonal bars) controlled the textures by exchanging the orientations of figure and ground regions (Lamme, 1995; Poort et al., 2016). Previous studies have provided a series of evidence that indicated independent encoding of FG and texture by analyzing natural images and their silhouette images (Kim et al., 2019; Pasupathy et al., 2019; Yamane et al., 2020b; Kimura et al., 2022; Kodama et al., 2022). Furthermore, despite the number of textures in our stimulus set is no more than a few hundreds, we consider that this would reasonably smooth out the effect of texture. Because of these reasons, we defined the FG modulation without the control of textures. To estimate the retinotopic position and extent of a CRF, we fitted a two-dimensional Gaussian function to a map of the responses to grating patches shown on a 5 × 5 grid (see the examples of estimated CRF in Supplementary Figure 3A). The CRF centers were not always close to the center of stimulus patches or distributed to a specific orientation (Supplementary Figure 3B). Twelve square-wave grating patches with different cycles (2, 4, 8 cycles) and orientations (0, 45, 90, and 135°) were used to estimate the center and extent of the CRF. The grating patches were 0.5 or 1° in diameter and arranged in a 5 × 5 grid covering a viewing angle of 3–12°. The total number of grating patterns was 300, and each patch was presented 10 times in each session in a pseudo-random order. The radius of the CRF was defined as 1 SD of the estimated function. The eccentricity of the CRF centers ranged between 2 and 10°. The positions for stimulus presentation were determined based on the estimated CRFs. In every recording session, the stimulus sizes were scaled to cover the CRFs, more than three times larger than the rough estimates of the CRF diameters (stimulus size: 2.5–21°). The scaling was inevitable to evoke neural responses, though the difference from the psychophysical experiment was introduced. Note also that the stimulus patches were not rotated in the electrophysiological experiment, in contrast to the psychophysical experiment. Our supplementary psychophysical experiment indicated that the effect of rotation was minimal (refer to Supplementary Figure 1). We labeled each stimulus “F” or “G” for each neuron based on whether figure or ground region (see how to decide figure and ground of each stimulus in section “2.4.2. Single-cell neural consistency”) was projected on the CRF center, i.e., a stimulus whose figure region was projected onto the CRF center of the neuron was given an “F” label. To examine responsiveness to visual stimuli, we compared the firing rates of single units for all stimuli (105 stimuli × 2 (original and mirror) × 10 repeats) during the prestimulus period (–50 to +50 ms from stimulus onset) and the stimulus period (+50 to +250 ms) by *t*-tests. *P* < 0.05 was used as the criterion for responsiveness. To examine FG modulation of single neurons, a one-way repeated-measures analysis of variance (ANOVA) was performed; we compared the spike counts of the stimulus period for all F stimuli (# of F stimuli × 10 repeats) with that for all G stimuli (# of G stimuli × 10 repeats). The percentage of FG neurons was 18% (71/387, *p* < 0.05). 83% (59/71) of FG neurons showed figure-preference, which was consistent with Poort et al. (2016). To convert a neural response that follows a Poisson distribution to a Gaussian distribution, we transformed the spike counts using the Anscombe transform, which transforms data with a Poisson distribution to an approximate Gaussian distribution, and performed the same analysis. The results did not differ from those without the transformation, as reported previously (Yamane et al., 2020b).

In the section “3.4. Correlations with neural modulation: Greater modulation with EASY stimuli” where the magnitudes of FG modulation for individual neurons were examined (refer also to section “2.4.4. Differential neural responses to FG”), we selected neurons based on the differential responses of a neuron to the original-mirror pair of a single stimulus rather than pooling the responses to F and G regions across stimulus patches. Since the FG labels depended on the combination of the stimulus patch and the CRF center, some pairs shared the same FG label for a particular neuron; there were pairs where the CRF center was located on the F (or G) region in both original and mirror images. Such pairs were excluded from the ANOVA that examined the significance of FG modulation. The percentage of the significant neurons was 16% (61/387, *p* < 0.05). The number of significant neurons was similar between the analyses based on the paired (61) and unpaired (71; described above) responses. 65% (40/61) of neurons were significant with both analyses. The neurons with the significant paired-responses (the responses were significantly different between the mirrored patches wherein F and G regions were projected onto the CRF, respectively) were subjected to the analysis in the section “3.4. Correlations with neural modulation: Greater modulation with EASY stimuli”.

### 2.4. Data analysis

#### 2.4.1. Perceptual consistency and reaction time

We quantified how consistently a region of a stimulus was perceived as figure across trials and participants. We defined the PC of a stimulus as the ratio of responses in the direction that was determined as figure across trials and participants as follows:


$$P\,C_{i}=\frac{1}{N_{s}}\,\sum_{j=1}^{N_{s}}\frac{\max\left(a_{i\,j},b_{i\,j}\right)}{N_{v}}\times 100.$$

Here, *N_v_* is the number of trials including 8 variants [2 (original and mirror) × 2 (left-right reversals) × 2 (presentation orders)] for an individual stimulus, *N_s_* is the number of participants (10), and *a*_*ij*_ and *b*_*ij*_ are the numbers of answers to each region (figure/ground), respectively, for stimulus *i* by participant *j*. PC for a single stimulus was computed from a total of 80 trials (8 trials × 10 participants). Note that PCs were computed for 105 different stimuli; it did not distinguish the eight variants because all variants have the same local properties such as texture, convexity, closedness, and symmetry. PC ranges from 50 to 100%, with 50 and 100% indicating the chance rate and perfect consistency, respectively. High consistency (or low ambiguity) in FG determination is considered to indicate ease of FG judgement. FG perception was not sensitive to the orientation (horizontal and vertical) of a figure against the background or the slight difference in the size and eccentricity of stimuli (see the detail in Supplementary Figure 1).

Mean reaction time (RT) across trials and participants for a single stimulus was calculated as follows:


$$R\,T_{i}=\frac{1}{N_{s}}\,\sum_{j=1}^{N_{s}}\frac{1}{N_{v}}\,\sum_{k=1}^{N_{v}}R\,T_{i\,j\,k}$$

where, RT_*ijk*_ is the reaction time and *k* is the index for repetitions (8 trials).

#### 2.4.2. Single-cell neural consistency

To examine the consistency of neural signals in response to FG organization in stimuli, we defined neural consistency (NC) as a correct rate for FG determination. For instance, if a neuron showed greater responses to all stimuli with the F label than to those with the G label, these responses were completely separable to F and G by a threshold, and thus the correct rate was 100%, i.e., the responses of this neuron were perfectly consistent (NC = 100%) in FG determination. In contrast, if a neuron showed random responses, the correct rate was chance (50%) and inconsistent (NC = 50%). We defined the NC of an individual neuron as the ratio of trials with correct responses as follows:


$$N\,C=\frac{\#\,T\,r\,i\,a\,l\,s_{c\,o\,r\,r\,e\,c\,t}}{\#\,T\,r\,i\,a\,l\,s_{a\,l\,l}}\times 100$$

where, #*Trials*_*correct*_ is the number of trials with correct responses (greater spike counts than the threshold for the preferred region or below the threshold for the non-preferred region), and #*Trials*_*all*_ is the total number of trials.

We set a threshold to distinguish between responses to figure and ground, and considered responses to be correct if a preferred region (F or G) of a patch caused a stronger-than-threshold response (true positive) or if a non-preferred region caused a weaker-than-threshold response (true negative). The threshold was determined for each neuron by support vector machine (SVM) so as to obtain the maximum correct rate. Although the threshold could be determined by a thorough search, SVM tends to show the optimal threshold with better generalization (the threshold is expected to show the best performance for other stimuli that were not shown). Since the NC values for all stimuli were computed by SVM in the previous study (Yamane et al., 2020b), their results can be compared directly with the present results computed by the same method. We used RBF (radial basis function) kernel SVM in the LIBSVM library^2^ as the classification model. The hyper parameters of the model were chosen automatically using the Grid Search Method implemented in the library. The training labels given to SVM were F and G (figure and ground, respectively, were projected onto the CRF center). The assignment of F/G labels (veridical FG labels) was provided based on the responses of all participants in the psychophysical experiments; that is, the region that was answered more often as figure across trials and participants was assigned F label (refer to section “2.2. Psychophysical experiment”). We used human-marked contours in the BSD. The human-marked contours were obtained by human tracking of object contours in natural images. We assigned FG labels to the regions that were segmented based on the contour. Specifically, we labeled both sides of the contour passing through the center of the image patch, so that only two segments existed in the most cases. In a few cases with a complex contour configuration, there existed a third region where FG label was not assigned. This region was assigned “non-FG” label. If the CRF center was located in this non-FG region, we excluded the data from the analysis. Using these training labels and the numbers of spikes from all trials, we estimated the threshold of the spike counts for F/G for each neuron to obtain the maximum correct rate. No cross-validation was performed since the classification was one dimensional with a relatively small number of data.

In this study, the NCs were calculated for two groups of stimuli, one with perceptually consistent stimuli and the other with ambiguous stimuli. We defined 20 stimuli with the highest and lowest PC values as the EASY (consistent) and HARD (ambiguous) stimulus groups, respectively. Although NC could be evaluated for individual stimuli, we decided to pool trials within each stimulus group for the sake of increasing signal-to-noise ratio. In the electrophysiological experiments, each stimulus had an original/mirror variant (2) and a repeat (10). The NC for each stimulus group was computed from a total of 400 trials (20 stimuli × 2 (original and mirror) × 10 repeats). Note that the original and mirror patches were treated as distinct patches since the spatial locations of the figure were different between the two patches. The thresholds were independently determined for each stimulus group to assure the best performance for each group. We excluded neurons from the analysis if the number of trials with valid spikes was 4 or less, resulting 81.7% (58/71) of the FG neurons analyzed for NC. The results of the analysis in the section 3.2 were not significantly dependent on this threshold. There was no significant difference between EASY and HARD stimulus group in the luminance contrast (permutation test, *p* = 0.22). The luminance contrast between figure and ground in EASY and HARD stimulus groups were 0.071 (SD = 0.045, median = 0.061) and 0.085 (SD = 0.063, median = 0.069), respectively. Note that the luminance values were computed by the rgb2gray function in MATLAB.

#### 2.4.3. Population-based neural consistency

To examine whether the collective activities of FG neurons contribute to the perception of FG, we computed a correct rate for FG classification from the activities of multiple neurons. We defined population-based neural consistency (PNC) as the correct rate for FG classification as follows:


$$P\,N\,C=\frac{\#\,T\,e\,s\,t\,P\,a\,t\,c\,h\,e\,s_{c\,o\,r\,r\,e\,c\,t}}{\#\,T\,e\,s\,t\,P\,a\,t\,c\,h\,e\,s_{a\,l\,l}}\times 100$$

where, #*TestPaches*_*correct*_ is the number of correctly classified test patches by responses of neural population, and #*TestPatches*_*all*_ is the total number of test patches.

We used SVM for the classification of FG from the activities of multiple neurons. The SVM model was identical to that used in Yamane et al. (2020b), and was an extension of that used in the computation of NC to multiple neurons. The SVM was trained with the responses of multiple neurons to EASY_50_ or HARD_50_ stimulus group. In this analysis, we defined 50 stimuli with the highest and lowest PC values as EASY_50_ and HARD_50_ stimulus groups, respectively. The number of stimuli was increased to 50 (compared to 20 used in the analysis with NC) for the sake of good convergence in learning. The SVMs were trained separately for the EASY_50_ and HARD_50_ stimulus groups in order to assure a fair comparison between their classification performances. This independent learning assured that the models were optimized for the individual stimulus groups, however, a mixed learning from all stimuli could generate a model with a biased performance for a particular stimulus group. The numbers of patches and trials in a learning set from a single neuron were 100 and 1,000, respectively (50 stimuli × 2 (original and mirror) × 10 repeats). Learning and testing of the machines were carried out with five-fold cross validation with random partitioning of data into the train and test data.

The SVM was trained to classify whether figure or ground was projected onto the CRF centers of neurons. Datasets for individual neurons were generated, and then combined to form the datasets for multiple neurons. First, the sum of spike counts over 10 repeated trials for each patch and its veridical label of FG were paired. Note that the veridical FG labels depended on the combination of the patch and the neuron. Because the location of the CRF centers differs across neurons, different veridical labels could be assigned to the same stimulus across neurons. Next, 80 patches (out of 100) with the veridical labels of F and G were sampled randomly without repetition to generate the dataset for a single neuron; a half of the 80 patches have F labels and another with G labels. Note that the dataset consisted of a list of spike counts and veridical labels but did not include patch identification, implying that the classification depended solely on the FG information. To classify FG from multiple neurons, the pre-determined number (1, 10, 20, 30, and 40) of neurons was randomly chosen without repetition across multiple recording sites and sessions. The dataset for a set of the chosen neurons was generated by combining the datasets of the single neurons to produce a *n* × *p* multidimensional data (*n*: the number of patches (80) with the F and G (40 + 40) labels; *p*: the number of neurons). The SVM was trained on 64 randomly selected patches out of 80 patches and the PNC was evaluated on the remaining 16 patches of test data (five-fold cross validation). This operation was repeated 200 times and the mean across them was defined as the PNC of that neuron set. Neurons with fewer responses (spikes were observed in ≦ 40 patches) to either figures or grounds were not taken into account, in order to assure complete datasets without missing data. With this selection of neurons, 64.8% (46/71) of the FG neurons were analyzed.

#### 2.4.4. Differential neural responses to FG

As an index to represent the magnitude of FG modulation of a single neuron, we computed the difference between the responses to the preferred and non-preferred regions (F or G). A greater difference is considered to indicate easier discrimination of FG. The differential neural responses to FG, △_*R*_, was defined as the difference between the numbers of spikes for the preferred and non-preferred regions of a single stimulus (the difference between the responses to the original and mirrored patches) as follows:


$${△}_{R_{i}}=R_{p\,r\,e\,f,i}-R_{n\,o\,n.p\,r\,e\,f,i}$$

where, *R*_*pref,i*_ and *R*_*non.pref,i*_ are the numbers of spikes for the preferred and non-preferred regions, respectively, for stimulus *i*. Stimuli were excluded from the analysis if original-mirror pair shared the same FG label or the pair did not evoke responses (refer to section “2.3.1. Neurons with FG-dependent responses” for the details).

#### 2.4.5. Modulation latency

To examine the relationship between the RT for FG judgement and the neural latency for FG modulation, we computed the modulation latencies for EASY and HARD stimulus groups (refer to section “2.4.2. Single-cell neural consistency”). First, an average peristimulus time histograms (PSTHs) across all FG neurons (71) and stimuli within a stimulus group were provided for the preferred and non-preferred regions (*R_pref_* (*t*) and *R_non.pref_* (*t*)). The difference between these PSTHs, *R_pref_* (*t*)−*R_non.pref_* (*t*), was defined as the average time course for FG modulation. A moving average was performed on the average time course, and then the latency for FG modulation was estimated as the intersection by a two-phase linear regression. The widths of the time window for the averaging was 50 ms and the range for the regression was 10–150 ms, respectively, which were empirically determined. Two-phase linear regression has a pair of linear models:


$$y_{0}=a_{0}\,x+b_{0}$$

$$y_{1}=a_{1}\,x+b_{1}$$

where, *x* are time points, *y* is the estimated time course for FG modulation, and *a* and *b* are the parameters of the linear models. The intersection of the lines is *r* = (*b*_1_−*b*_0_)/(*a*_1_−*a*_0_). Two-phase linear regression fits the pair of straight lines by minimizing the total sum of the squared deviations. The intersection, *r*, indicating the point in time at which the spike rate was modulated by FG, was defined as the estimated modulation latency. When the modulation latency was estimated by two-phase regression on the cumulative sum of the FG modulation series (Sugihara et al., 2011), the sensitivity was worse than ours, and the latency was estimated to be slightly longer. However, no difference in the conclusion was obtained.

#### 2.4.6. Statistical test

To evaluate statistical significances of the difference of two variables in analysis of single-cell neural consistency and population-based neural consistency (i.e., NC for EASY/HARD and PNC for EASY_50_/HARD_50_), we used non-parametric permutation test instead of any parametric tests because these distributions did not follow a Gaussian distribution. A null distribution was created by shuffling the data 10,000 times.

## 3. Results

We investigated the correlation between the PC and the modulation of neural signals in response to FG in order to clarify whether FG neurons in V4 contribute to the perception of FG. Psychophysical studies have reported that a number of stimuli showed a relatively low level of consistency in FG judgement for natural image patches across participants and trials (Fowlkes et al., 2007; Sakai et al., 2015), suggesting that the participants were able to correctly judge FG for some stimuli but not others. Although Yamane et al. reported that approximately one-quarter of the neurons showed significant FG modulation (Yamane et al., 2020b), most of individual neurons exhibited a low consistency in FG determination across stimuli, suggesting that the neurons were capable of correctly estimating FG for some stimuli but not others. We hypothesized that the PC in FG judgment widely varies across stimuli, which reflects the reliability of neural modulation evoked by the stimulus. Specifically, some stimuli evoke reliable modulation for FG neurons and yield consistent FG judgements, and other stimuli do not evoke sufficient neural modulation and yield inconsistent judgements. We estimated the PC of single patches in human psychophysical experiments with the same natural patches as those used in the neural recordings and then examined the correlations between the PC and the consistency, magnitude, and latency of neural modulation in FG determination.

### 3.1. Perceptual consistency for FG determination in local natural images

**FIGURE 3:**
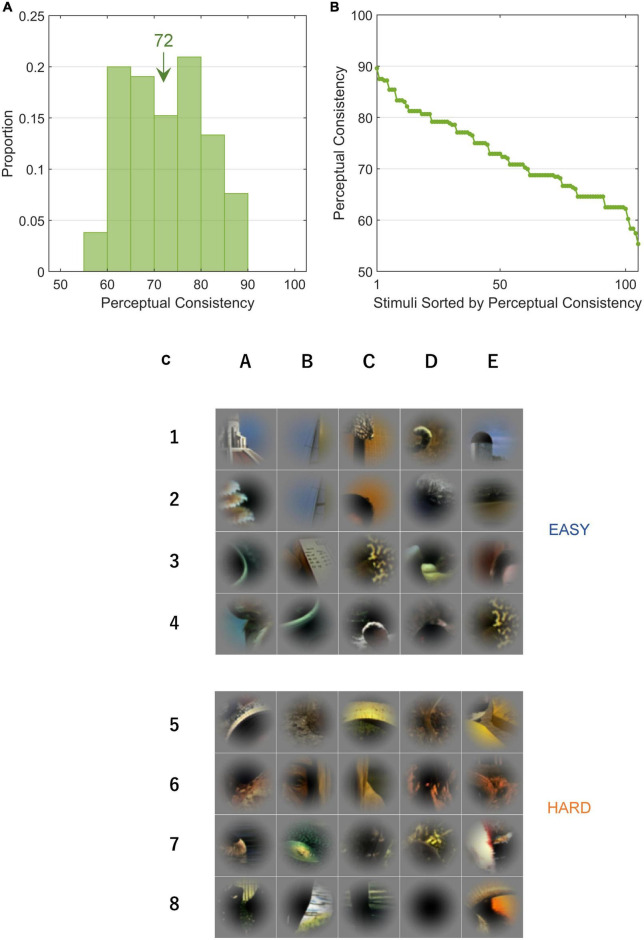
Perceptual consistency and EASY/HARD stimuli. PC is an index that indicates the ease with which a figure was perceived. **(A)** The distribution of PC for all stimuli. The inset value indicates the mean. **(B)** PCs in rank order. **(C)** The top and bottom panels (rows 1–4 and 5–8) show EASY and HARD stimuli, respectively (20 original stimuli with the highest and lowest PCs, respectively). Unlike Figure 1, the patches were not rotated.

We performed psychophysical experiments to examine how consistent human FG judgments are for single local natural images by repeatedly testing the DoF with 80 trials (10 participants × 8 variants). We measured the PCs for 105 local images which included a variety of shapes, textures, and colors. PC = 100% indicates that all participants consistently answered the DoF through all trials and 50% indicates the chance (refer to section “2.4.1. Perceptual consistency and reaction time”). A few patches appear similar to each other because they were extracted from the same natural image but from different parts (Figure 1). With these patches, their figure regions were sometimes different because the attributes of the contours were different and their PC values were relatively low. In some patches, a face region was not considered figure because of the inversion of hue and direction which had substantial effects in face detection and recognition (George et al., 1999; Minami et al., 2015). The distribution of measured PCs for all stimuli is shown in Figure 3A. The PC values in rank order are shown in Figure 3B, indicating a Gaussian-like distribution (approximately Gaussian distribution with mean = 72.3%, SD = 8.0%; one-sample Kolmogorov-Smirnov test, *p* = 0.202). The stimuli with the 20 highest and 20 lowest PC values (EASY and HARD stimulus groups used for NC, respectively; refer to section “2.4.2. Single-cell neural consistency”) are shown in the top and bottom panels, respectively, in Figure 3C. Consistent with previous studies (Ramenahalli et al., 2014; Sakai et al., 2015), prominent convexity and occlusion tended to evoke a high level of PC, and other factors, such as differences in texture and color, also appeared to contribute to PC. In the following sections, we examined the properties of neural modulation in relation to PC.

### 3.2. Correlations with single-cell neural consistency: Greater consistency with EASY stimuli

We examined the hypothesis that FG neurons show more consistent FG determination with the EASY stimulus group that evoked consistent FG perception than with the HARD stimulus group that evoked ambiguous perception. 83.1% (59/71) of FG neurons showed the preference for figures (F-preferring neurons). We estimated the NC of the FG neurons whose number of valid trials (the number of spikes > 0) was 5 or greater (58 neurons; refer to section “2.4.2. Single-cell neural consistency” for details) with the EASY and HARD stimulus groups. NC was defined as the ratio of trials with the correct FG determination (refer to section “2.4.2. Single-cell neural consistency” for details). For instance, if a neuron showed greater responses to all stimuli with F labels compared to stimuli with G labels, the responses of this neuron were perfectly consistent (NC = 100%). We set a threshold that differentiates responses to F from those to G and considered responses to be correct if a preferred region (F or G) of a patch caused a stronger-than-threshold response (true positive) or if a non-preferred region caused a weaker-than-threshold response (true negative), resulting in the range of NC between 50 and 100%. Higher and lower levels of NC were expected with the EASY and HARD stimulus groups, respectively. The mean NCs for the 24 neurons among the nearest, intermediate, and farthest from the fovea were 55, 54, and 56%, respectively, indicating the eccentricity independence of NC at least within the examined range (2–10°).

**FIGURE 4:**
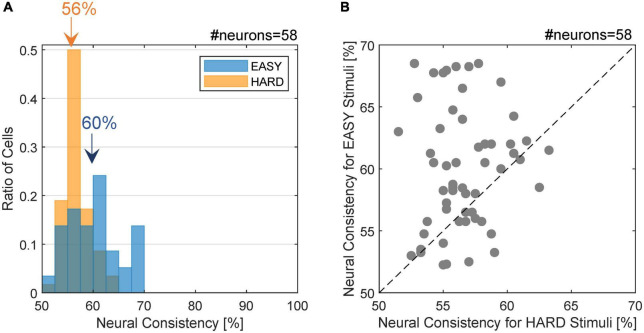
Distribution of neural consistency with EASY/HARD stimuli. **(A)** The histogram of NC for the EASY/HARD stimuli (blue/orange) across FG neurons. The inset values indicate the means for the EASY and HARD stimulus groups. **(B)** The distribution of NCs. The dots indicate individual neurons. Those located above the dotted diagonal line are the neurons with higher NC for EASY group compared to HARD group, which constitute 72.4% of the neurons examined.

The distribution of NC for the FG neurons is shown in Figure 4A. The NC with the EASY stimulus group appeared to be slightly greater than those with the HARD stimulus group. The mean NCs with the EASY and HARD stimulus groups were 60.0% (SD = 4.9%) and 56.6% (SD = 2.5%), respectively, and the difference was significant (one-sided permutation test; *p* < 10^–4^). The ratio of the neurons with the same FG preference as the preference which used all stimuli was 97.0% (32/33) for EASY stimuli and 83.3% (15/18) for HARD stimuli. The denominator was the number of FG neurons with significance to each stimulus set. The distribution of NCs for individual neurons is shown in Figure 4B wherein 72.4% (42/58) of FG neurons showed a greater NC for EASY stimulus group than for HARD stimulus group. The correlation between the NCs for EASY and HARD groups was low (Pearson’s product-moment coefficient, *r* = 0.08). These results indicate that a number of FG neurons responded more consistently to stimuli that evoked more consistent FG perception.

### 3.3. Correlations with population-based neural consistency

In the previous section, we compared PC and single-cell NC. Although a significant difference in NC was observed between EASY and HARD stimulus groups, the difference was less than that in PC. Population coding would account for this low correlation between the NC and PC. A previous study reported that the single-cell NC was no more than slightly above the chance rate but the integration of the responses from multiple FG neurons substantially increased the correct rate in FG classification to the degree similar to the perception (Yamane et al., 2020b). The difference in the correct rates between EASY and HARD stimulus groups are expected to increase to the degree similar to the difference in PC.

**FIGURE 5:**
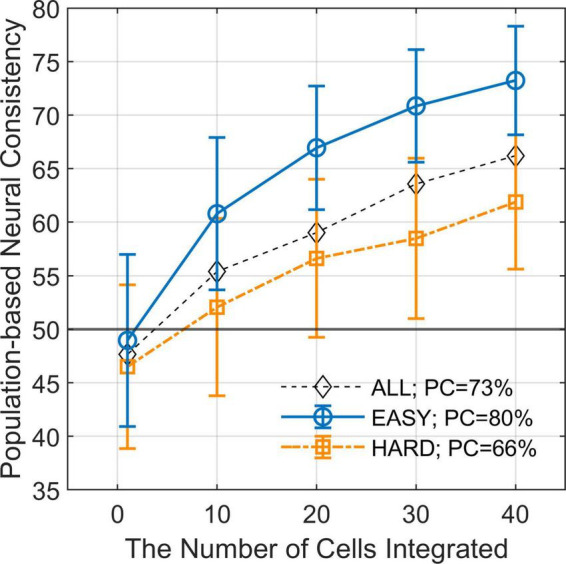
Population-based neural consistency as a function of the number of cells integrated. The estimated PNCs (mean across simulations) as a function of the number of integrated neurons for EASY_50_ stimuli (blue circles), HARD_50_ stimuli (orange squares), and for all stimuli (black diamonds). The error bars indicate SDs. The simulation was repeated 200 times for every condition with randomization. The difference in PNC between EASY stimuli and HARD stimuli increases as the number of integrated neurons increases when FG neurons were pooled.

We estimated PNC from the responses of multiple FG neurons and examined the difference in PNC between EASY_50_ and HARD_50_ stimulus groups which included the stimuli with fifty highest and lowest PCs, respectively (refer to section “2.4.4. Differential neural responses to FG” for details). The estimated PNCs are shown in Figure 5 as a function of the number of neurons integrated. The mean PNCs computed from all stimuli increased from the chance level (47.7% with SD = 8.11%) for a single neuron to 66.2% (SD = 6.45%) for 40 neurons. Note that the PNC for a single neuron is slightly smaller than the NC in the previous section because PNC was computed with cross validation. As the number of neurons increased, the difference in PNC between EASY_50_ and HARD_50_ groups increased. The differences were statistically significant (one-sided permutation test, *p* < 10^–4^) for the entire range. The difference was 11.4% with 40 neurons (PNCs were 73.2 and 61.8% for EASY_50_ and HARD_50_ groups, respectively), which was similar to the difference in PC (14.0%; 79.7 and 65.7% for EASY_50_ and HARD_50_ stimuli, respectively). This result suggests that the responses of a few tens of FG neurons include the information necessary for FG perception.

### 3.4. Correlations with neural modulation: Greater modulation with EASY stimuli

**FIGURE 6:**
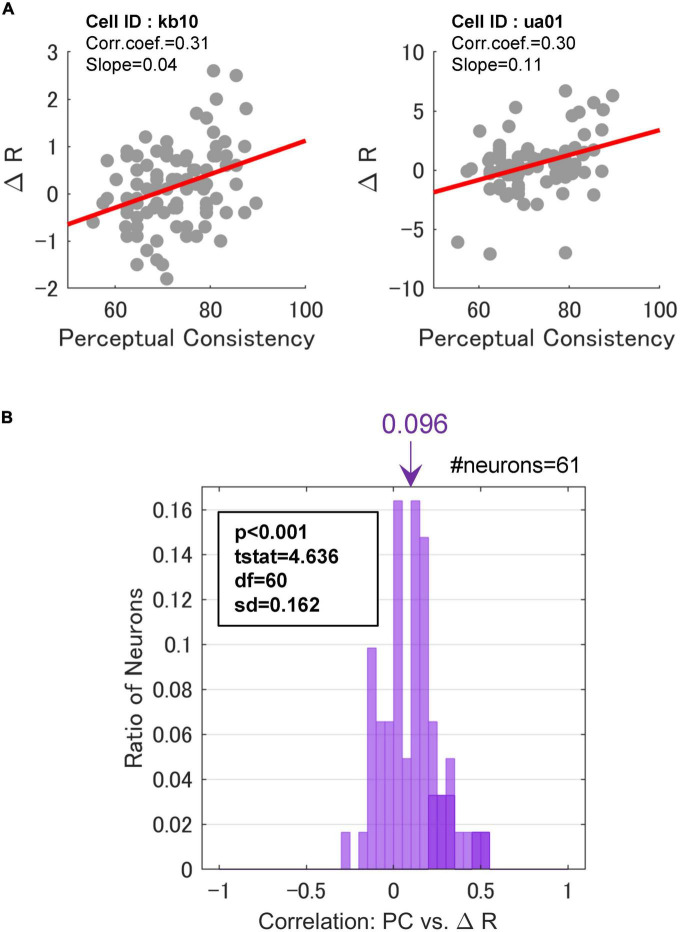
Correlations between perceptual consistency and ΔR. **(A)** PC and △_*R*_ in a scatter plot for two example FG neurons. Dots represent individual stimuli. The red lines indicate the linear regression. **(B)** The distribution of Spearman’s rank correlations between PC and △_*R*_. The inset indicates the results of a one-sided *t*-test against 0. Neurons with significant correlations were colored darker.

We examined the hypothesis that FG neurons show greater FG modulation with the EASY stimulus group that evoked consistent FG perception, compared to the HARD stimulus group. Specifically, we expected to observe a positive correlation between PC and the magnitude of neural FG-modulation which was represented by the difference between the neural responses to the preferred and non-preferred regions, △_*R*_. The regression coefficients between PC and △_*R*_ for single neurons were estimated. The results of two example neurons are shown in Figure 6A, wherein we observed a positive correlation between PC and △_*R*_. The Spearman’s rank correlations of these two neurons were approximately 0.3, and their regression coefficients were positive and significant (*p* < 10^–3^). These neurons showed a tendency that larger FG modulation was evoked by the stimuli that were easier for FG perception.

Across the neurons with the significant paired responses (the responses were significantly different between the mirrored patches wherein F and G regions were projected onto the CRF, respectively; refer to section “2.3.1. Neurons with FG-dependent responses” for the detail), 70.5% (43/61) of neurons showed positive regression coefficients, and 13.1% (8/61) showed significance (*t*-test, *p* < 0.05). In contrast, 29.5% (18/61) of neurons showed negative coefficients, although none of these neurons showed significance. This result indicates that the degree of FG modulation in at least some neurons showed a positive correlation with the ease of FG perception. The distribution of the rank correlations is shown in Figure 6B. The distribution appears to be biased toward the positive end. The mean rank-correlation was 0.096 (SD = 0.162), which was significantly larger than zero (one-sided *t*-test; *t*(60) = 4.64, *p* < 10^–3^). This result indicates that the mean modulation of the neurons was slightly larger with the EASY stimulus group that evoke consistent FG perception than with the HARD stimulus group. A similar result was observed when FG neurons without the significant paired responses were included in the analysis. These results indicate that neural FG modulation was positively correlated with consistency in FG perception, i.e., FG neurons in monkey V4 showed larger FG modulation for natural patches with easier FG perception. We have also performed the same analysis with the normalized △_*R*_, and the same conclusion was drawn (Supplementary Figure 4).

### 3.5. Correlations between perceptual reaction times and neural latencies: Shorter reaction times and latencies with EASY stimuli

**FIGURE 7:**
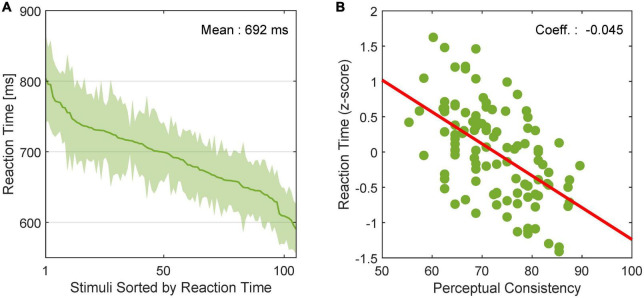
Reaction time of human FG perception. **(A)** RT for FG perception in rank order. The solid line and shade indicate the mean and SE, respectively, across participants. **(B)** Relationship between PC and RT in *z* score. Dots represent individual stimuli. The red line indicates the linear regression line.

We examined the relationship between the dynamics of FG perception and neural FG modulation. Stimuli included in EASY stimulus group were expected to evoke shorter RTs for FG perception and shorter modulation latencies in FG neurons than those in HARD stimulus group. First, to examine whether RTs were shorter for stimuli that were easier for FG perception, we estimated the mean RTs across participants for individual stimuli. The mean RTs in rank order are shown in Figure 7A with the standard error (SE). We observed a wide range of RTs depending on stimuli. The relationship between the RTs in z-score and PCs is shown in Figure 7B. The Pearson’s product-moment coefficient was –0.53, and the regression coefficient was –0.045 (*p* < 10^–3^), indicating that the RT was shorter for stimuli with easier FG perception (greater PC).

**FIGURE 8:**
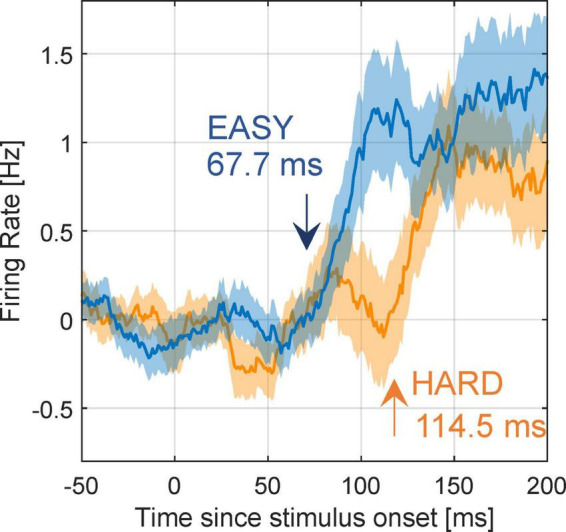
Modulation latency with EASY/HARD stimuli. The differential time courses for the EASY/HARD stimulus groups. Blue and orange lines indicate the mean time courses for EASY and HARD stimulus groups, respectively, across all FG neurons, and shades indicate SEs. Two-phase regression was applied to these time courses to compute modulation latencies. The inset values are the estimated modulation latencies.

Next, we examined whether the modulation latencies of FG neurons were shorter with EASY stimulus group than with HARD stimulus group. The mean time series of FG modulation, *R*_*pref*_(*t*)−*R*_*non*.*pref*_(*t*), across FG neurons (71) are shown in Figure 8 (refer to section “2.4.5. Modulation latency” for details). The modulation latencies estimated from the mean time series were 67.7 and 114.5 ms with the EASY and HARD stimulus groups, respectively, indicating a shorter modulation latency with the EASY stimulus group than with the HARD stimulus group. The PSTHs of example neurons that support the slower modulation latency to HARD stimulus group are shown in Supplementary Figure 5. These results suggest that the time necessary for processing FG was longer for stimuli with more difficult FG perception than for easy stimuli; a consistent relationship between the dynamics of FG perception and neural FG modulation.

We examined whether other factors than PC could evoke the relation between the PC and modulation latency. First, to exclude a possibility that the observed modulation latency was the reflection of the visual response latency that could depend on other factors than PC, we examined the visual response latencies of the preferred and non-preferred stimuli for EASY and HARD stimulus groups. The measured visual response latencies were approximately 60 ms independent of the stimulus conditions, indicating that the modulation latency did not depend on the visual response latency (refer to Supplementary Figure 6). Next, to clarify whether the difference in modulation latency was not influenced by the response magnitude of individual neurons, we examined the mean time series of FG modulation from the population PSTHs with magnitude normalization. To obtain the normalized PSTHs for individual neurons, we normalized the responses by the greatest response with the subtraction of baseline firing rate between –50 and +50 ms. These PSTHs were averaged over neurons to obtain the normalized population PSTH. The modulation latencies measured from the normalized population PSTHs for EASY and HARD stimulus groups were 72.1 and 115.2 ms, respectively (Supplementary Figure 7). These latencies were similar to those from the unnormalized PSTHs (67.7 and 114.5 ms, respectively), indicating that FG modulation latency did not depend on the response magnitude of individual neurons. The analyses on the correlations between the perceptual consistency/ambiguity and the reliability of neural modulation in the determination of FG with a variety of local natural images support that V4 neurons with FG-dependent responses contribute to FG perception.

## 4. Discussion

We investigated whether the ambiguity of FG perception correlated with the reliability of FG modulation in V4 neurons in response to local natural images. First, to quantify the ambiguity of FG perception, we performed a psychophysical experiment using the same stimuli as the previous electrophysiological experiment (Yamane et al., 2020b) and defined PC for each stimulus. The PC was widely distributed across the stimulus set, enabling us to examine the correlation with the reliability of FG modulation. The results showed that stimuli with higher PCs, in comparison with stimuli with lower PCs, evoked significantly greater magnitudes in NC and neural modulation, and shorter RTs and neural latencies. These results indicate substantial correlations between human FG perception and the responses of FG neurons in V4, suggesting that FG neurons in V4 contribute to the perception of FG segregation.

Natural stimuli contained multiple visual features that contribute to FG perception. We considered the ease of FG perception for individual local natural stimuli based on the consistency in perception across trials and participants. The ease of FG perception seemed to be determined by a combination of factors including convexity and closedness of contour, texture, and color. For instance, a mixture of a highly convex contour, a large difference in texture, and high contrast appear in EASY stimuli (refer to Figure 3C). Meanwhile, some HARD stimuli include a highly convex contour or a large difference in texture, which is likely denying the dominant influence of a single factor but supporting the substantial role of the combination of multiple factors. The integration of these visual features appears to contribute to the establishment of FG perception in natural images. Electrophysiological studies comparing the responses of single neurons to shape and texture have suggested that these visual features were separately encoded by distinct populations in V4 (Kim et al., 2019; Machida et al., 2021; Kimura et al., 2022; Kodama et al., 2022). Lateral interactions among V4 neurons through intrinsic connections (Yoshioka et al., 1992) are likely to contribute to the integration of multiple features in FG perception. However, we still do not know how V4 neurons extract and integrate multiple features in natural images for FG segregation. Studying the representation and integration of multiple features would contribute to the further understanding of FG perception.

Here we discuss alternative explanations in which the responses of FG neurons are modulated by other visual features rather than by FG. First, when a boundary is included in the CRF, it is also possible that the modulation depends on the location of the boundary than FG. Such an issue has been discussed in the previous studies; e.g., responses of V1 neurons to texture figure (Rossi et al., 2001) and V4 neurons for which relatively large CRFs did not allow to keep the boundaries outside of the CRF (Zhou et al., 2000; Poort et al., 2012). Previous works on FG modulation in V1 neurons reported a greater number of F-preferring neurons even when the location of the contours were identical (e.g., Poort et al., 2016). Although the locations of contours were not identical in our stimulus set, the tendency of the greater number of F-preferring neurons was reproduced. In our experiment, 83% of FG neurons showed F-preference. The locations of boundary were likely independent of the neural modulation originated from FG. Second, the FG neurons could prefer a combination of the convexity and direction of a contour with respect to the CRF center (such as C-shaped contour located on the left). Since natural objects tend to have convex contours, if the CRF center was located near the figure center, the convex contour pointing away from the CRF center could be an effective clue for detecting figure. However, our stimuli were local images cropped out of natural scenes and often did not include the figure (object) center. The directions of contours with respect to the CRF center were often strongly biased from that with respect to the figure center. Contours in our stimuli are sometimes concave in the direction away from the CRF center. The combination of the convexity and the direction of contour would not be a reliable clue in local natural images. Additionally, in our stimulus set, the orientation and convexity of the contour passing through the stimulus center were uniformly distributed across stimuli. Furthermore, we also controlled the distribution of closedness. Therefore, we consider that the responses of FG neurons were indeed modulated by FG.

Although we designed the stimulus presentations in the electrophysiological and psychophysical experiments as similar to each other, they differed in dimension, rotation, and presentation location, which might limit the comparison between the neural modulation and perceptual consistency. In the psychophysical experiments, the stimuli with a fixed dimension of 6° × 6° were presented at a fixation position with rotation such that the border between figure and ground were rotated to the vertical. In contrast, during the electrophysiological experiments, the stimuli were scaled and positioned at and around the CRFs of the neurons in recording in order to evoke reasonable responses of the neurons. The range of the eccentricity across recorded neurons was 2–10° which was similar to those reported in previous recordings from V4 [e.g., 0.0–6.6° (Pasupathy and Connor, 2002), 1–12° (Motter, 2009)]. The dimensions of the presented stimuli, which evoked reasonable responses with the given eccentricity, was 9.1 ± 4.3°(mean ± SD), which roughly approximated the stimulus dimension in the psychophysical experiments (6°). To consider the similarity in the stimulus presentations, the spatial positions and dimensions of the CRFs with respect to the patch center might also be useful indices. Since the stimuli were shown at a fixation point in the psychophysical experiments, it might be ideal if the CRF centers were located within the stimuli and uniformly distributed over the stimuli and if the CRF extents were covered by the stimuli. In the present experiments, all CRF centers were located within the stimuli (refer to Supplementary Figure 3). The distance between the stimulus and CRF centers was 1.3 ± 1.2°, and the equivalent diameter of the CRF extents was 2.3 ± 2.1°. Given these ranges, a quarter to the entire stimulus often covered the single CRFs. Although the determination of the appropriate correspondence in stimulus dimension and position to psychophysical experiments depends on multiple factors and is not straightforward, the similarity between our electrophysiological and psychophysical experiments might fall onto a reasonable range. Previous studies have shown scale invariance in the responses to curvatures (El-Shamayleh and Pasupathy, 2016) and objects (Rust and DiCarlo, 2010) in V4 neurons. The border ownership-selective neurons in V2 and V4 also showed scale invariance (Zhou et al., 2000). It might be reasonable to expect that FG responses in V4 neurons also show scale invariance, which would ease the requirement of the similarity in stimulus presentation. However, there might be limitations in the comparison between the electrophysiological and psychophysical data. Electrophysiological recordings on awake monkeys during a behaviorally relevant task are expected to contribute to the better comparison between the neural responses and perception.

In the present study, the correlations between PC and the reliability of FG-dependent responses of single neurons were statistically significant but moderate. This result is consistent with Yamane et al. (2020b) where single FG neurons showed a weak ability in FG discrimination for natural image patches. They also reported that the integration of the responses of dozens of FG neurons enabled the correct discrimination across a variety of patches, indicating a crucial role of population coding. We introduced the same integration method as Yamane et al. (2020b) and evaluated the population-based neural consistency, PNC. As the number of integrated neurons increased, the difference in PNC between EASY and HARD stimuli increased. These results support the contribution of a population of FG-dependent neurons to FG perception.

Although the visual response latency of FG neurons was unchanged, their FG modulation latency was slower for the stimuli that were difficult to perceive FG, indicating that the time necessary for FG determination differs depending on the difficulty of FG segregation. This result is consistent with the dependence of modulation latency in V4 for curvature detection on the degree of occlusion over the stimulus (i.e., ambiguity of shape information) (Kosai et al., 2014). The predictive coding suggested that this occlusion-dependent delay was due to the contribution of feedback from higher areas such as the prefrontal cortex (Choi et al., 2018). Kar et al. have also reported the dependence of modulation latency on the difficulty in object recognition and the crucial role of feedback (Kar et al., 2019). Recurrent processing in neural circuits responsible for FG segregation would account for the long modulation latencies for images with ambiguous FG perception.

Stability of FG perception also provides insights into the recurrent processing. Bistable (ambiguous) perception, compared to unambiguous perception, activates higher brain regions and dramatically enhances top-down influence (Wang et al., 2013). The top-down processing by recurrent connections appears to account for a delay observed in the ambiguous perception. Parkkonen et al. suggested that the percept-related activity in the early visual cortex reflected the top-down influence that maintained the FG segregation when a stimulus with ambiguous FG was presented (Parkkonen et al., 2008). In the determination of border ownership in ambiguous images, Wagatsuma et al. suggested that BOS neurons in V2 with the opposite preferences compete for a short duration, and then they are modulated by top-down influence and establish stable perception (Wagatsuma et al., 2008). F- and G-preferring neurons in V4 are expected to similarly respond to an ambiguous stimulus through a feed-forward computation which may compete through lateral connections, followed by top-down influence that breaks the balance to stabilize. With our HARD stimuli, the competitions might need to be regulated and stabilized by recurrent connections which enabled a transition to a state in which one of the regions was determined figure, so that we observed a longer latency in the HARD stimuli. In contrast, if a stimulus was not ambiguous, no competition took place and the system was stabilized without top-down influence, and thus we observed a shorter latency in EASY stimuli. This competition model is also consistent with the psychological phenomenon of not being able to perceive both sides of a contour as figures at the same time. The competition model with recurrent processing is likely to account for the difference in the modulation latency. A comparison between the collective response of FG neurons and the perception of FG would lead to a better understanding of the neural basis of FG perception and the recognition of natural scene.

## Data availability statement

Publicly available datasets were analyzed in this study. This data can be found here: http://hdl.handle.net/11094/73784 (Data associated with publication population coding of figure and ground in natural image patches by V4 neurons. Osaka University Knowledge Archive. doi: 10.18910/73784).

## Ethics statement

The studies involving human participants were reviewed and approved by Research Ethics Committee for Faculty of Engineering, Information and Systems, University of Tsukuba (# 2020R375). The participants provided their written informed consent to participate in this study. The animal study was reviewed and approved by Osaka University Animal Experiment Committee (certification no: FBS-13-003).

## Author contributions

MS performed psychophysical experiment. MS and KS analyzed data. All authors wrote and edited the manuscript and contributed to the conception and design of the study and approved the submitted version.

## Footnotes

1
http://www.nbrp.jp

2
https://www.csie.ntu.edu.tw/~cjlin/libsvm/
