# Supplementary material for: Correlation between neural responses and human perception in figure-ground segregation
Source: Front Syst Neurosci. 2023 Jan 12;16:999575. doi: 10.3389/fnsys.2022.999575 (PMC9877615; doi:10.3389/fnsys.2022.999575)
Supplement: Supplementary file 1 [file Data_Sheet_1.pdf]

Supplementary Material

Correlation between Neural Responses and Human Perception in Figure-Ground Segregation

Motofumi Shishikura*, Hiroshi Tamura, Ko Sakai

*** Correspondence:** Ko Sakai: sakai@cs.tsukuba.ac.jp

1.1 Supplementary Figures

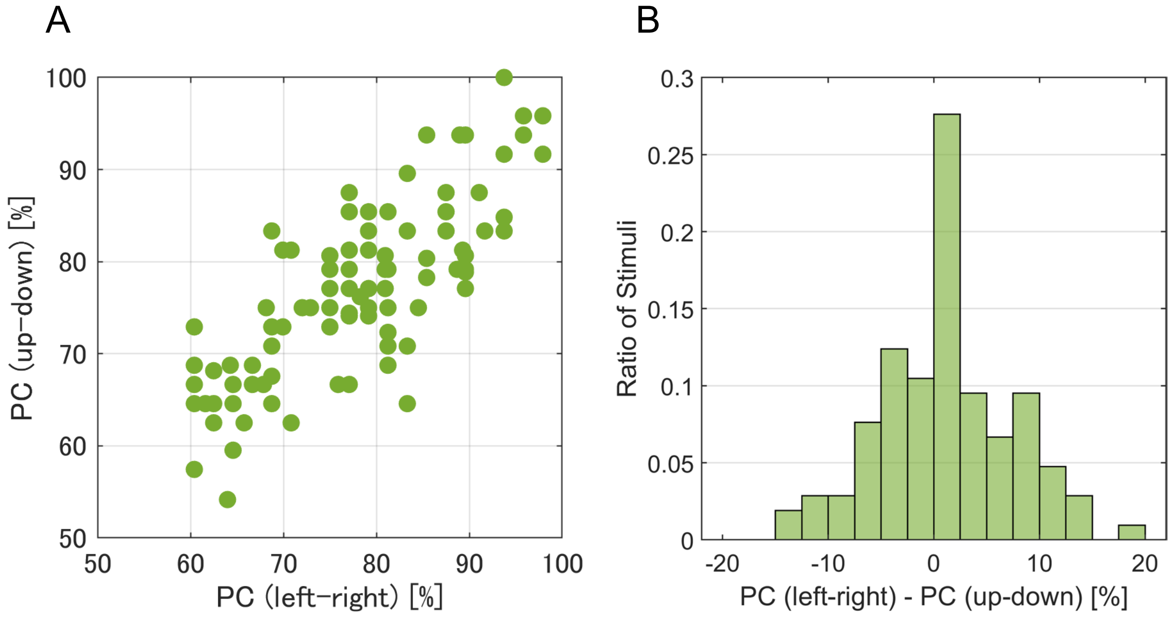

Supplementary Figure 1. Perceptual consistencies (PC) measured from left-right 2-alternative forced choice (2AFC) and up-down 2AFC. To clarify whether the orientation of our local image stimuli affects PC, we performed an additional psychophysical experiment with 6 participants. The difference between the additional and original experiments was only orientation which was the horizontal (180°; up-down 2AFC) and vertical (0°; left-right 2AFC) for additional and original, respectively. (A) Relationship between PCs measured from the left-right 2AFC and up-down 2AFC. The measured PCs for the two orientations were strongly correlated (r = 0.80). (B) The histogram of the difference between the PCs measured from the left-right and up-down 2AFCs with the standard deviation of 6%.
To clarify the effect of the size and location of stimuli on PC, we also performed another psychophysical experiment with the stimuli whose size and eccentricity were identical to those used in the electrophysiological experiments (size = 9° × 9°, eccentricity = 5°; the mean size and eccentricity across all electrophysiological recordings). The measured PCs were fairly correlated with the original PCs (left-right; Pearson's product-moment coefficient = 0.74).

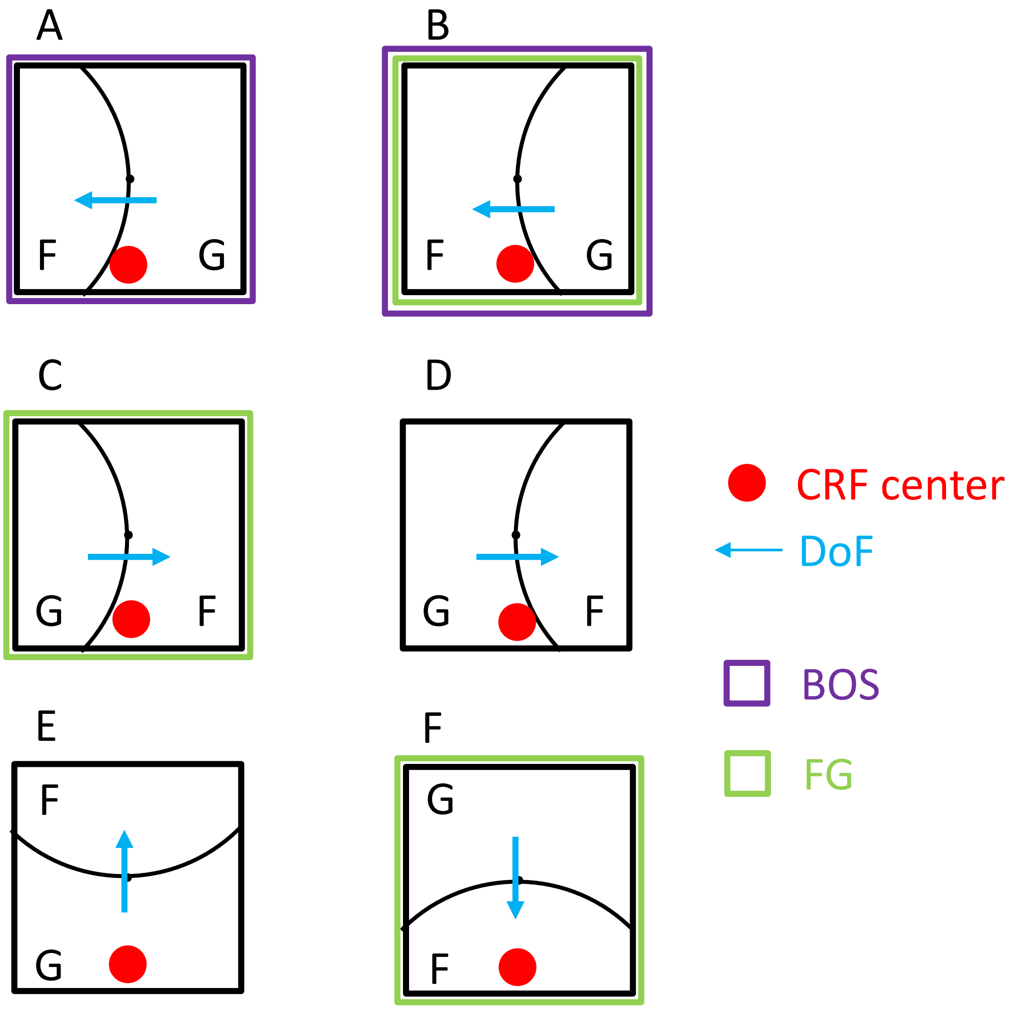

Supplementary Figure 2. The difference between BOS and FG modulation. (A and B) The panels show the stimuli with a figure region on the left with respect to the contour. If the neuron is border ownership selective (Zhou, et al., 2000) and prefers the direction of figure (DoF) on the left, this neuron responds significantly to these stimuli. (B and C) The panels show the stimuli with a figure region projected onto the CRF center. If the neuron is figure-ground (FG) significant and prefers Figure, this neuron responds significantly to these stimuli. Therefore, with the stimulus in B, border ownership selectivity (BOS) and FG modulations cannot be distinguished. FG neurons do not always encode BOS. In the present analysis, we analyzed FG modulation but not BOS, though FG and BOS modulations are not independent. Furthermore, we controlled the orientation of the contour passing through the stimulus center so that the orientations were equally distributed. (E and F) There are stimuli in which the orientation of the contour at the center is horizontal. FG modulation is observed in these stimuli as well while the BOS modulation is not (the preferred DoF does not match with the DoFs of the stimuli).

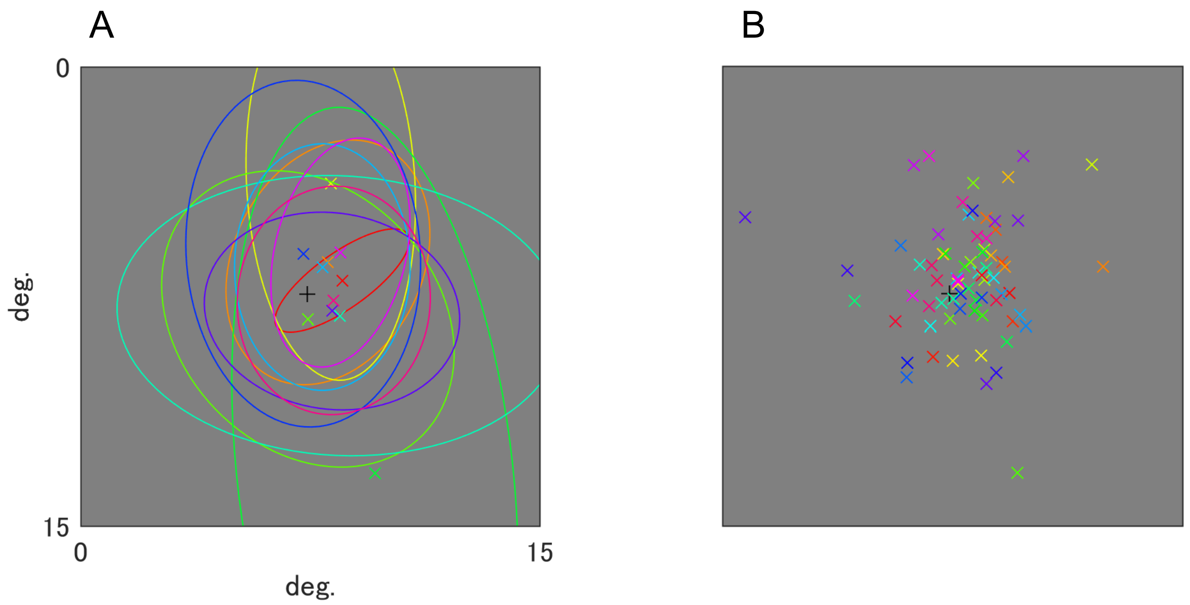

Supplementary Figure 3. The estimated classical receptive fields (CRFs) of FG neurons. **(**A) The estimated classical receptive fields (CRFs) of FG neurons in an example recording session. The CRF centers (crosses) and extents (eclipses) were estimated by fitting a 2D Gaussian to the neural responses. (B) The CRF centers of FG neurons (71) across all recording sessions. The grey square (panel) indicates the normalized extent of stimuli. In every recording session, stimuli were scaled to cover the CRFs of the neurons. In this panel, we normalized the stimulus size to draw the CRF centers across sessions. The contour remained passing through the center of this panel.

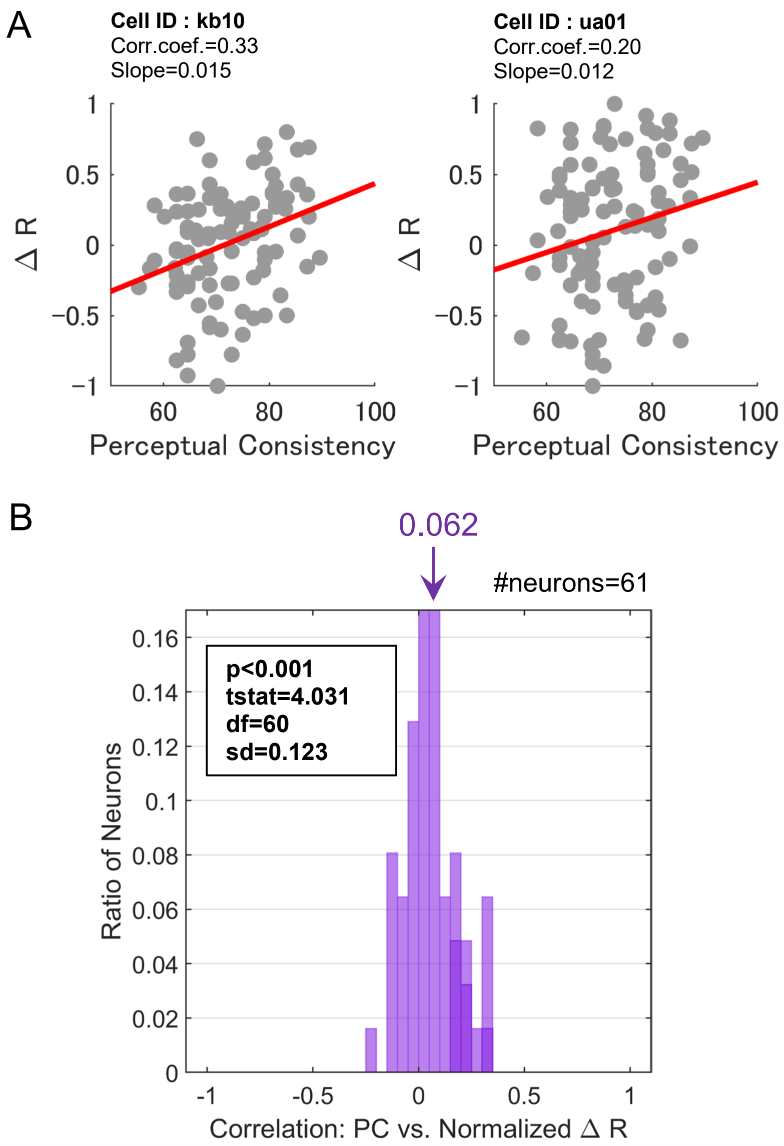

Supplementary Figure 4. Correlations between perceptual consistency and Δ_R with normalization. The normalized $\Delta_{R_i} = \frac{R_{pref,i} - R_{non.pref,i}}{R_{pref,i} + R_{non.pref,i}}$ was calculated, which ranges from -1 to +1. (A) PC and Δ_R in scatter plots for two example FG neurons. Dots represent individual stimuli. The red lines indicate the linear regression. (B) The distribution of Spearman's rank correlations between PC and Δ_R. The inset indicates the results of a one-tailed t-test against 0. For the normalized Δ_R, 69% (42/61) of neurons showed positive regression coefficients between PC and Δ_R, and 12% (6/61) showed significance (p<0.05). In contrast, 29% (19/61) of neurons showed negative coefficients, and none of them showed significance. The mean rank-correlation between PC and Δ_R was 0.063 (SD: 0.12), which was significantly larger than zero (one-sided t-test; $t(60) = 4.03$, $p < 10^{-3}$). These results were consistent with the results computed from the Δ_R without normalization as described in the main text (Figure 6).

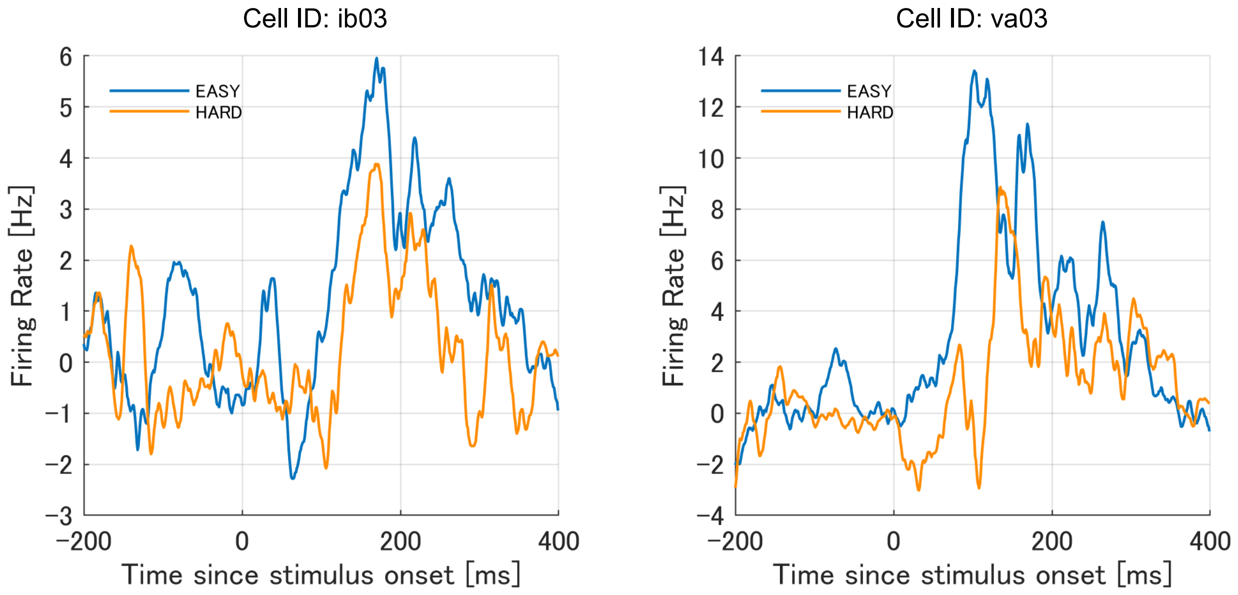

Supplementary Figure 5. Peristimulus time histograms (PSTHs) of two example neurons that support the slower modulation latency for HARD stimulus group. Blue and orange lines indicate the mean differential time courses of PSTH for EASY and HARD stimulus groups.

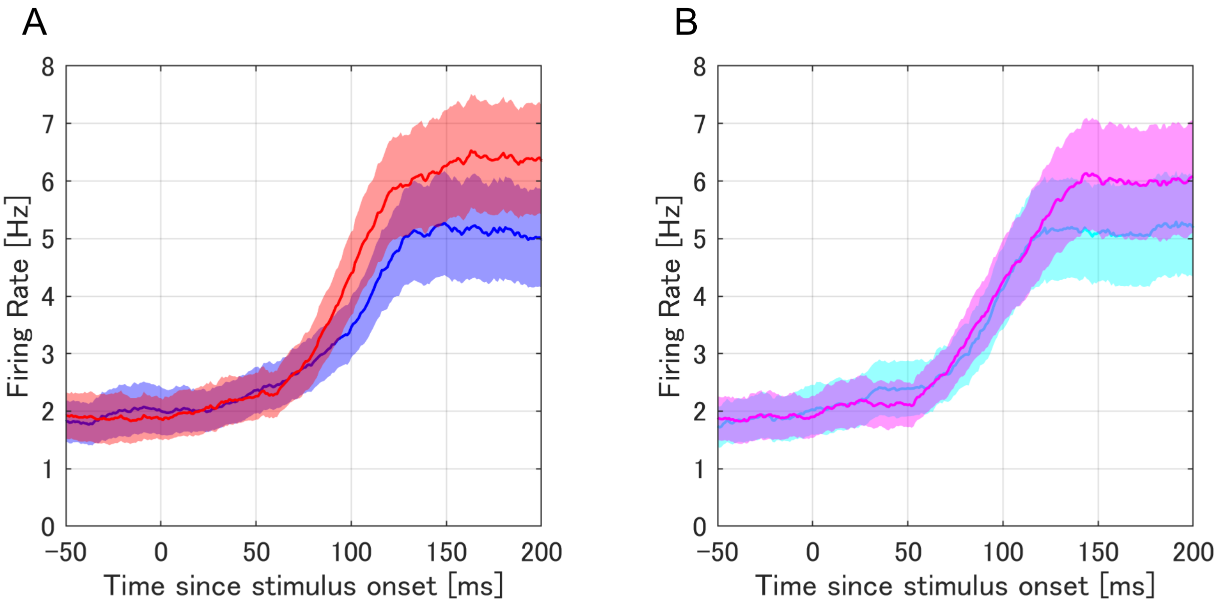

Supplementary Figure 6. The population PSTHs for EASY and HARD stimulus groups. (A)The population PSTHs to the preferred (red line) and non-preferred (blue line) stimuli for EASY stimulus group across all FG neurons. Shades indicate SEs. (B) The population PSTHs to the preferred (magenta line) and non-preferred (cyan line) stimuli for HARD stimulus group across all FG neurons.

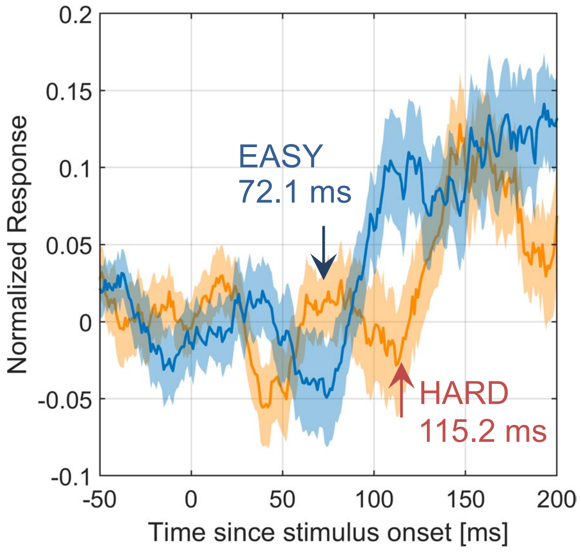

Supplementary Figure 7. Mean modulation latencies across FG neurons for EASY/HARD stimuli computed from the normalized response. The responses were normalized by the maximum response of the neuron, and then pooled to draw a PSTH by the two-phase regression analysis (refer to "2.4.5 Modulation latency" in the main text for details). Blue and orange lines indicate the mean differential time courses for EASY and HARD stimulus groups, respectively, with shades indicating SE. The inset values show the estimated modulation latencies.
